# Supplementary material for: Tracheal aspirate RNA sequencing identifies distinct immunological features of COVID-19 ARDS
Source: Nat Commun. 2021 Aug 26;12:5152. doi: 10.1038/s41467-021-25040-5 (PMC8390461; doi:10.1038/s41467-021-25040-5)
Supplement: Supplementary file 1 — Supplementary Information [file 41467_2021_25040_MOESM1_ESM.pdf]

Supplementary Information: “Tracheal aspirate RNA sequencing identifies distinct immunological features of COVID-19 ARDS”

Supplementary Results

Comparison against external datasets

We compared our findings against three publicly available RNA-seq datasets from studies of SARS-CoV-2 infection[1–3]. Given that no prior lower respiratory fluid transcriptomic studies have focused specifically on patients with ARDS, the most relevant comparison was with respect to our COVID-ARDS versus No-ARDS analysis, although we also compared against our COVID-ARDS versus Bacterial LRTI-ARDS analysis, which had a larger sample size.

We first assessed our findings against a study of post-mortem lung tissue from COVID-19 or control patients[1] and identified overlapping differentially expressed genes (Supplementary Data 12a) related to chemokine signaling, type 1 interferon signaling and toll like receptor signaling pathways, which were upregulated in the COVID-19 groups (Supplementary Data 12b). We subsequently compared our results to a study evaluating BAL gene expression in a rhesus macaque model of SARS-CoV-2 infection[2] and also identified overlapping differentially expressed genes with respect to our COVID-ARDS versus No-ARDS analysis (Supplementary Data 12c). Functional enrichment of the genes upregulated SARS-CoV-2 infection identified pathways related to interferon signaling, coronavirus pathogenesis and cytokine signaling (Supplementary Data 12d).

In addition, we evaluated our data against a recently published BAL transcriptional profiling dataset of COVID-19 patients and controls with or without pneumonia[3] and identified overlapping differentially expressed genes (Supplementary Data 12e) representing pathways including interferon-gamma signaling and SARS-CoV-2 innate immunity evasion in the patients with COVID-19 (Supplemental Data 12f). We also found significant overlap of differentially expressed genes with respect to the COVID-19 vs bacterial pneumonia comparison in this study and our COVID-ARDS versus Bacterial-LRTI ARDS analysis (Supplementary Data 12g). Functional enrichment analysis demonstrated that shared differentially expressed genes

upregulated in COVID-19 patients across both studies represented pathways including the host anti-viral response (Supplementary Data 12h) and those upregulated in patients with bacterial pneumonia in both studies represented IL-1, TLR and myeloid cell activation pathways (Supplementary Data 12i). We note that these external studies did not exclude COVID-19 patients receiving immunosuppressants, a criterion that we imposed to ensure transcriptional profiling results (in particular those related to immune signaling) most accurately reflected the underlying biology of disease.

Comparison against matched TA and mini-BAL samples

Because bronchial alveolar lavage (BAL) fluid has been used more frequently than TA to study the lower respiratory tract of COVID-19 patients[2–5], we compared in silico cell type deconvolution of RNA-seq data from eight subjects with matched TA and mini-BAL specimens from a previously published study in the control cohort[6]. No significant differences in cell type proportions were observed, suggesting that TA had the potential to comparably assesses the lower respiratory tract transcriptional environment of patients with pneumonia (Supplementary Data 13, Supplementary Figure 7).

Supplementary Figures

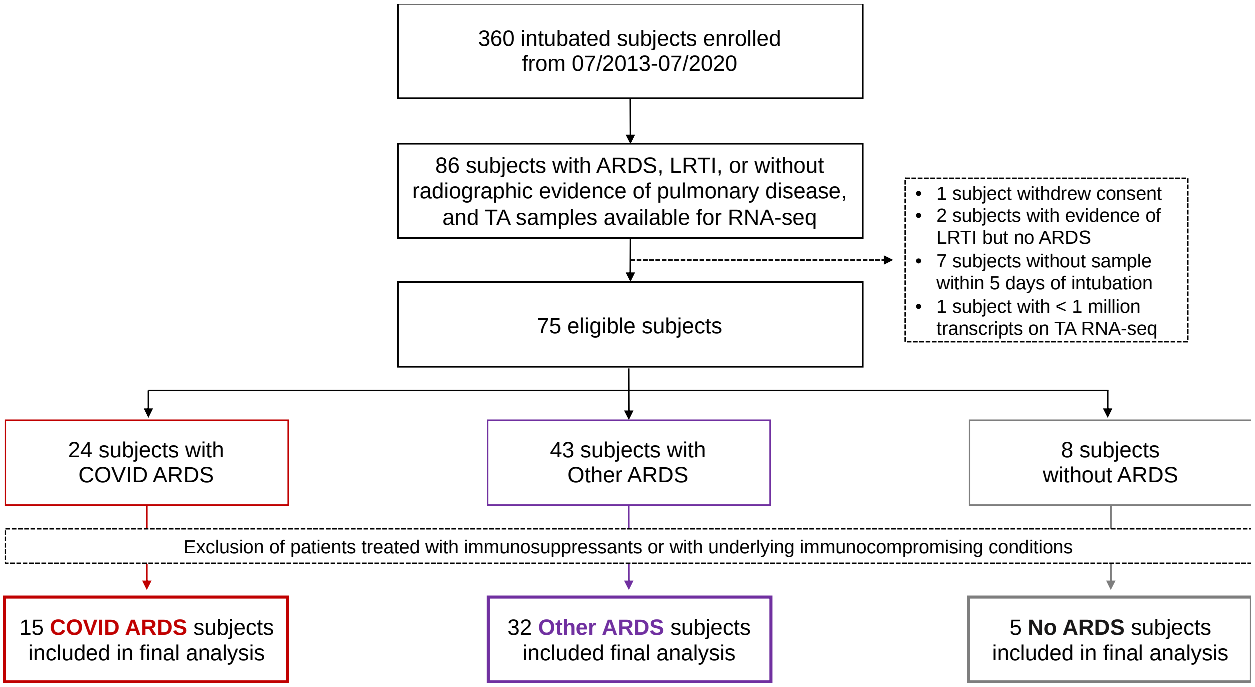

Supplementary Figure 1. **CONSORT diagram depicting patient enrollment.** We analyzed all subjects with TA specimens available for RNA-seq who either: 1) had clinically adjudicated acute respiratory distress syndrome (ARDS) due to other viral, bacterial, or non-infectious etiologies (COVID-ARDS, Other-ARDS groups), or who were intubated for airway protection without radiographic evidence of pulmonary pathology (No-ARDS group). Of the 360 enrolled subjects, 86 had TA samples available for RNA-seq. Subjects who withdrew consent (n=1), who had LRTI but no ARDS (n=2), who did not have a TA sample collected within five days of intubation (n=7), or who had TA samples yielding < $1x10^6$ protein-coding transcripts on RNA-seq (n=1), were excluded. Of the remaining 75 eligible subjects, those treated with immunosuppression or with underlying immunocompromising conditions were excluded, leaving 15 COVID-ARDS, 32 Other-ARDS and five No-ARDS subjects for final analysis.

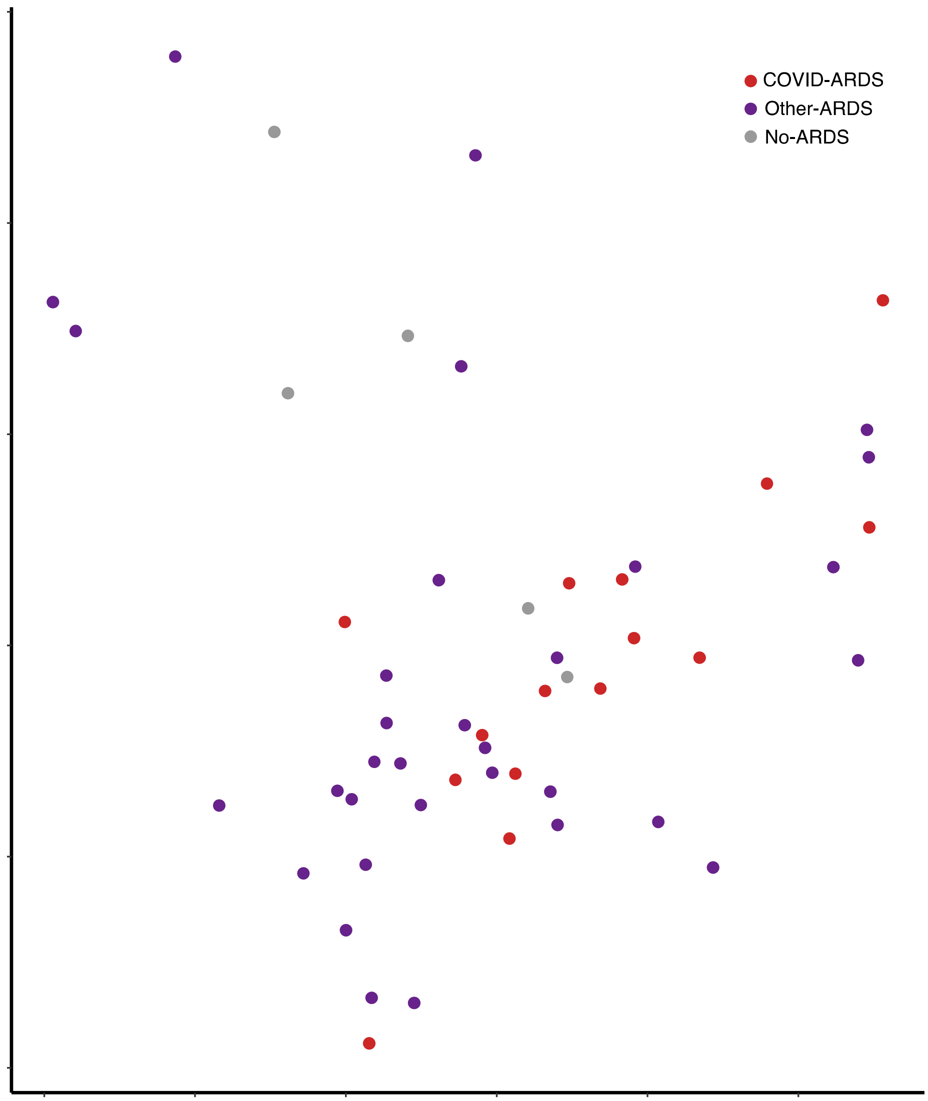

Supplementary Figure 2. **Principal component analysis of gene expression in TA samples.** The plotPCA function in DESeq2 was used to analyze the 500 genes with the highest variance in TA samples from **in TA samples from the COVID-ARDS, Other-ARDS and No-ARDS groups.**

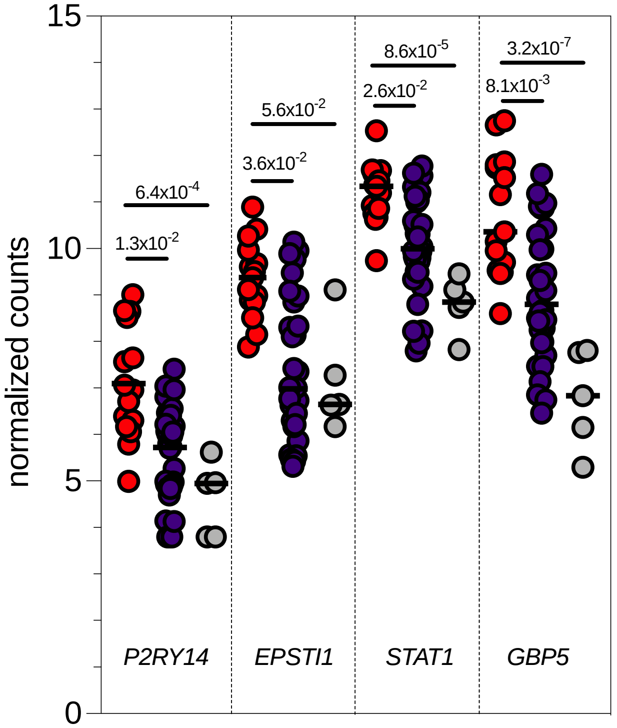

Supplementary Figure 3. **Normalized expression of genes differentially expressed between COVID-ARDS and Other-ARDS groups also predicted to be most inhibited by both dexamethasone and G-CSF.** Normalized expression for COVID-ARDS (red, n=15), Other-ARDS (violet, n=32), and No-ARDS controls (grey, n=5) were compared using a two-sided Wald test in DESeq 2. Adjustments for multiple comparisons were made using the independent hypothesis weighted Benjamini-Hochberg adjusted P-value, which is indicated above the bar spanning each comparator group. Complete list of genes inhibited or activated by dexamethasone, G-CSF and other drugs computationally predicted by IPA to modulate the transcriptional response of COVID-ARDS against a comparator groups is found in Supplementary Data 6. Source data can be found in the Source Data file. Lines depict median values.

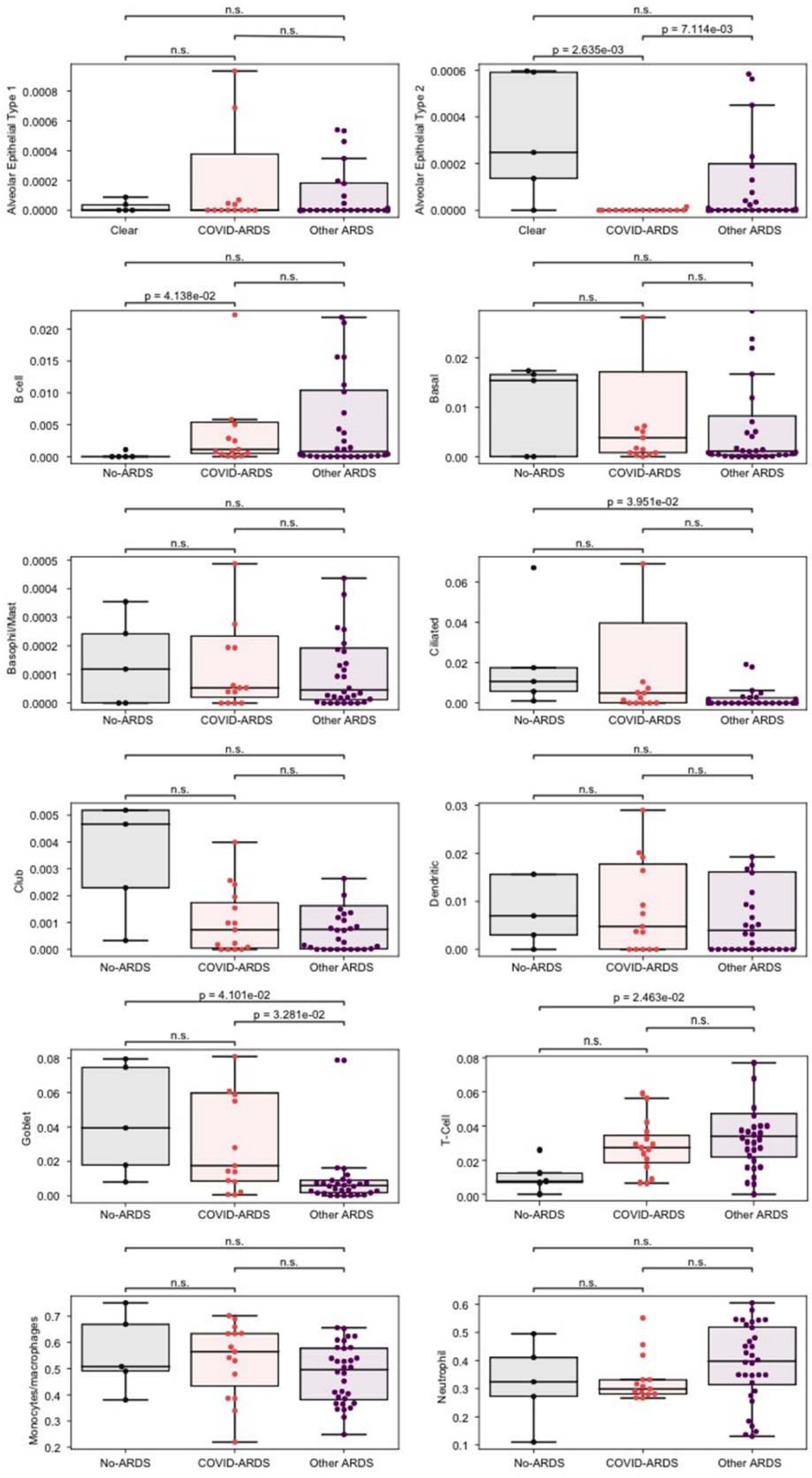
n.s.
n.s.
n.s.
Alveolar Epithelial Type 1
0.0008
0.0006
0.0004
0.0002
0.0000
Clear
COVID-ARDS
Other ARDS
n.s.
p = 7.114e-03
p = 2.635e-03
Alveolar Epithelial Type 2
0.0006
0.0004
0.0002
0.0000
Clear
COVID-ARDS
Other ARDS
n.s.
n.s.
p = 4.138e-02
B cell
0.020
0.015
0.010
0.005
0.000
No-ARDS
COVID-ARDS
Other ARDS
n.s.
n.s.
n.s.
Basal
0.02
0.01
0.00
No-ARDS
COVID-ARDS
Other ARDS
n.s.
n.s.
n.s.
Basophil/Mast
0.0005
0.0004
0.0003
0.0002
0.0001
0.0000
No-ARDS
COVID-ARDS
Other ARDS
p = 3.951e-02
n.s.
n.s.
Ciliated
0.06
0.04
0.02
0.00
No-ARDS
COVID-ARDS
Other ARDS
n.s.
n.s.
n.s.
Club
0.005
0.004
0.003
0.002
0.001
0.000
No-ARDS
COVID-ARDS
Other ARDS
n.s.
n.s.
n.s.
Dendritic
0.03
0.02
0.01
0.00
No-ARDS
COVID-ARDS
Other ARDS
p = 4.101e-02
p = 3.281e-02
n.s.
Goblet
0.08
0.06
0.04
0.02
0.00
No-ARDS
COVID-ARDS
Other ARDS
p = 2.463e-02
n.s.
n.s.
T-Cell
0.08
0.06
0.04
0.02
0.00
No-ARDS
COVID-ARDS
Other ARDS
n.s.
n.s.
n.s.
Monocytes/macrophages
0.7
0.6
0.5
0.4
0.3
0.2
No-ARDS
COVID-ARDS
Other ARDS
n.s.
n.s.
n.s.
Neutrophil
0.6
0.5
0.4
0.3
0.2
0.1
No-ARDS
COVID-ARDS
Other ARDS

Supplementary Figure 4. **In silico deconvolution of cell types from tracheal aspirate bulk RNA-sequencing data using lung single cell signatures.** The horizontal line inside the box denotes the median and the lower and upper hinges correspond to the first and third quartiles, respectively. Whiskers extend from the hinge to the largest (smallest, respectively) value no more than 1.5*IQR away from the hinge, where IQR is the interquartile range. The y-axis in each panel was trimmed at the maximum value among the three patient groups of 1.5*IQR above the third quartile. Pairwise comparisons between patient groups were performed with a two-sided Mann-Whitney-Wilcoxon test followed by Bonferroni's correction (n=15 COVID-ARDS, n=32 Other ARDS, n=5 No-ARDS). Data are tabulated in (Supplementary Data 7) and can be found in the Source Data file.

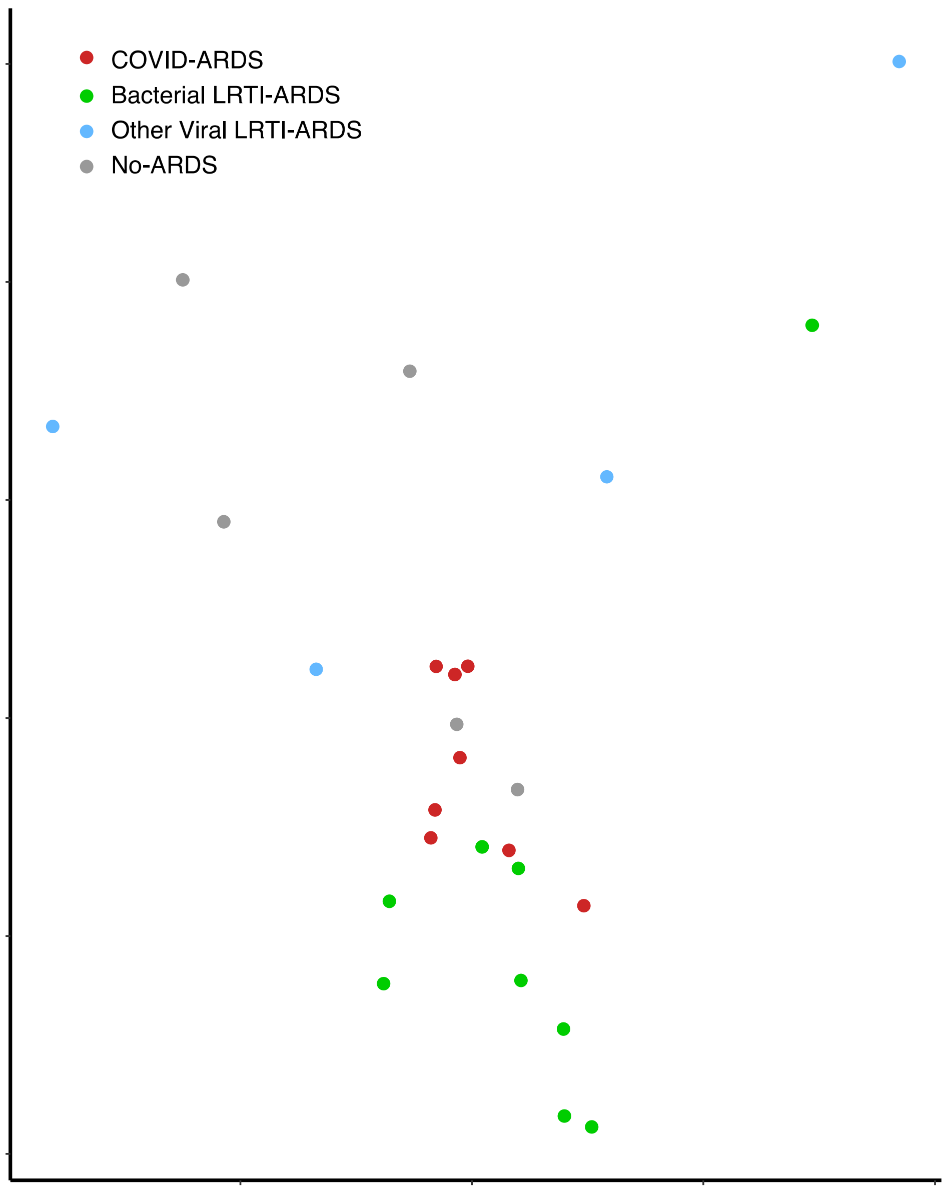

Supplementary Figure 5. **Principal component analysis of gene expression in samples used for subgroup analysis.** The plotPCA function in DESeq2 was used to analyze the 500 genes with the highest variance in TA samples from COVID-ARDS, Viral LRTI-ARDS, Bacterial LRTI-ARDS, and No-ARDS groups.

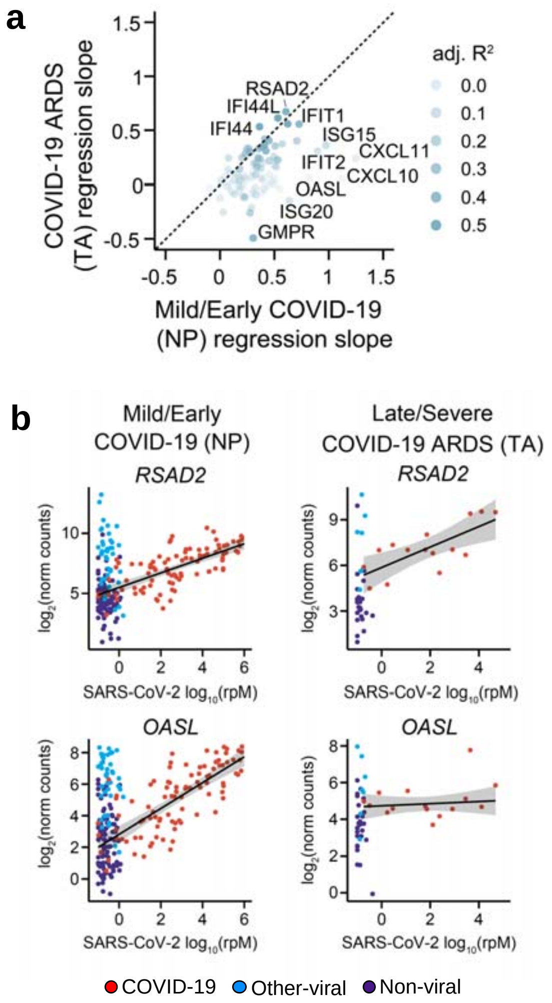

Supplementary Figure 6. **Relationship between SARS-CoV-2 viral load and interferon-stimulated gene (ISG) expression.** a) Scatterplot of the relationship between ISG counts and SARS-CoV-2 viral load (reads per million, rpM), quantified by the regression slope, in nasopharyngeal (NP) samples from patients with mostly mild/early COVID-19 (x-axis) and in tracheal aspirate (TA) samples from patients with severe COVID-19 and ARDS (y-axis). b) Scatter plots of normalized gene counts (log2 scale, y-axis) as a function of SARS-CoV-2 viral load (log10(rpM), x-axis). Robust regression was performed on SARS-CoV-2 positive patients. Lines represent the regression estimates and shaded bands represent 95% confidence intervals. RSAD2 is an ISG whose expression (y-axis) is correlated with SARS-CoV-2 viral load

(x-axis) in both early/mild (NP) and severe (TA) disease, while OASL is an ISG for which the correlation observed in early/mild COVID-19 is absent in severe COVID-19 patients with ARDS. Values are tabulated in (Supplementary Data 11) and can be found in the Source Data file.

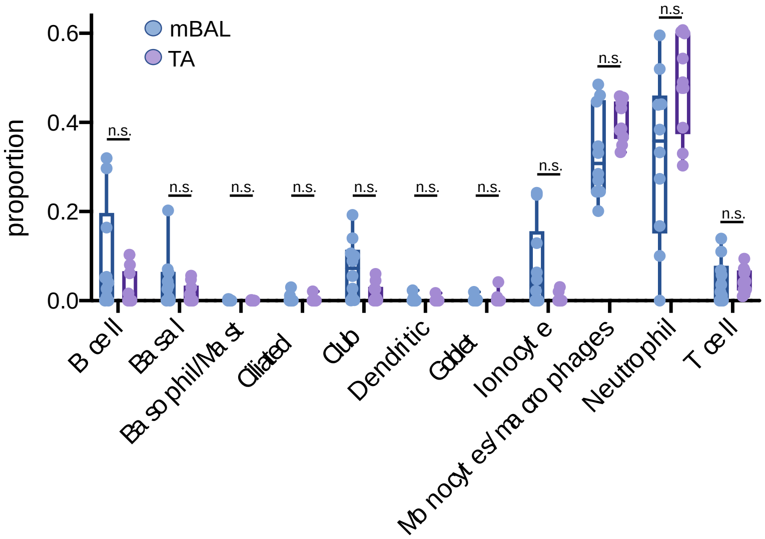

Supplementary Figure 7. **Cell type proportions in matched TA and mini-BAL samples.** In silico deconvolution of cell types using lung single cell signatures from matched TA and mini-BAL samples (n=8) from patients with bacterial LRTI in the control cohort that underwent bulk RNA-sequencing. The horizontal line inside the box denotes the median and the lower and upper hinges correspond to the first and third quartiles, respectively. Whiskers extend from the hinge to the largest (smallest, respectively) value no more than 1.5*IQR away from the hinge, where IQR is the interquartile range. The y-axis in each panel was trimmed at the maximum value among the three patient groups of 1.5*IQR above the third quartile. Pairwise comparisons between patient groups were performed with a two-sided Mann-Whitney-Wilcoxon test followed by Bonferroni's correction. Values are tabulated in (Supplementary Data 13) and can be found in the Source Data file.

Supplementary References

1. Ackermann, M. *et al.* Pulmonary Vascular Endothelialitis, Thrombosis, and Angiogenesis in Covid-19. *New England Journal of Medicine* **383**, 120–128 (2020).
2. Aid, M. *et al.* Vascular Disease and Thrombosis in SARS-CoV-2-Infected Rhesus Macaques. *Cell* **183**, 1354-1366.e13 (2020).
3. The NU SCRIPT Study Investigators *et al.* Circuits between infected macrophages and T cells in SARS-CoV-2 pneumonia. *Nature* **590**, 635–641 (2021).
4. Liao, M. *et al.* Single-cell landscape of bronchoalveolar immune cells in patients with COVID-19. *Nat Med* **26**, 842–844 (2020).
5. CONTAGIOUS collaborators *et al.* Discriminating mild from critical COVID-19 by innate and adaptive immune single-cell profiling of bronchoalveolar lavages. *Cell Res* **31**, 272–290 (2021).
6. Kalantar, K. L. *et al.* A Metagenomic Comparison of Tracheal Aspirate and Mini-Bronchial Alveolar Lavage for Assessment of Respiratory Microbiota. *American journal of physiology. Lung cellular and molecular physiology* (2019) doi:10.1152/ajplung.00476.2018.
7. Krämer, A., Green, J., Pollard, J. & Tugendreich, S. Causal analysis approaches in Ingenuity Pathway Analysis. *Bioinformatics* **30**, 523–530 (2014).
8. Mick, E. *et al.* Upper airway gene expression reveals suppressed immune responses to SARS-CoV-2 compared with other respiratory viruses. *Nature Communications* **11**, (2020).
9. Subramanian, A. *et al.* Gene set enrichment analysis: A knowledge-based approach for interpreting genome-wide expression profiles. *Proceedings of the National Academy of Sciences* **102**, 15545–15550 (2005).
10. Mi, H., Muruganujan, A., Ebert, D., Huang, X. & Thomas, P. D. PANTHER version 14: more genomes, a new PANTHER GO-slim and improvements in enrichment analysis tools. *Nucleic Acids Res* **47**, D419–D426 (2019).
