# Supplementary material for: Tracheal aspirate RNA sequencing identifies distinct immunological features of COVID-19 ARDS
Source: Nat Commun. 2021 Aug 26;12:5152. doi: 10.1038/s41467-021-25040-5 (PMC8390461; doi:10.1038/s41467-021-25040-5)
Supplement: Supplementary file 16 — Reporting Summary [file 41467_2021_25040_MOESM16_ESM.pdf]

nature research

Corresponding author(s): Charles Langelier
Last updated by author(s): Jun 24, 2021

Reporting Summary

Nature Research wishes to improve the reproducibility of the work that we publish. This form provides structure for consistency and transparency in reporting. For further information on Nature Research policies, see our Editorial Policies and the Editorial Policy Checklist.

Statistics

For all statistical analyses, confirm that the following items are present in the figure legend, table legend, main text, or Methods section.

n/a	Confirmed	
☐	☒	The exact sample size (*n*) for each experimental group/condition, given as a discrete number and unit of measurement
☐	☒	A statement on whether measurements were taken from distinct samples or whether the same sample was measured repeatedly
☐	☒	The statistical test(s) used AND whether they are one- or two-sided<br>*Only common tests should be described solely by name; describe more complex techniques in the Methods section.*
☐	☒	A description of all covariates tested
☐	☒	A description of any assumptions or corrections, such as tests of normality and adjustment for multiple comparisons
☐	☒	A full description of the statistical parameters including central tendency (e.g. means) or other basic estimates (e.g. regression coefficient) AND variation (e.g. standard deviation) or associated estimates of uncertainty (e.g. confidence intervals)
☐	☒	For null hypothesis testing, the test statistic (e.g. *F*, *t*, *r*) with confidence intervals, effect sizes, degrees of freedom and *P* value noted<br>*Give P values as exact values whenever suitable.*
☐	☒	For Bayesian analysis, information on the choice of priors and Markov chain Monte Carlo settings
☒	☐	For hierarchical and complex designs, identification of the appropriate level for tests and full reporting of outcomes
☐	☒	Estimates of effect sizes (e.g. Cohen's *d*, Pearson's *r*), indicating how they were calculated

Our web collection on statistics for biologists contains articles on many of the points above.

Software and code

Policy information about availability of computer code

Data collection	Clinical data were collected and stored using QuesGen and REDCap databases

Data analysis	Bulk RNAseq and differential expression: Statistical analysis was performed using R v4.0.3 and Bioconductor v3.12. Following demultiplexing, sequencing reads were pseudo-aligned with kallisto39 (v. 0.46.1; including bias correction) to an index consisting of all transcripts associated with human protein coding genes (ENSEMBL v. 99), cytosolic and mitochondrial ribosomal RNA sequences, and the sequences of ERCC RNA standards. Differential expression analysis was performed using DESeq2 1.32.0. Significant genes were identified using an independent-hypothesis-weighted, Benjamini-Hochberg false discovery rate (FDR) less than 0.142,43 using IHW v.1.20.0. Empirical Bayesian shrinkage estimators for log2-fold change were fit using apeglm v.1.14.0. Pathway analysis was performed using Ingenuity Pathway Analysis (March 2021 Release). Cell-type proportions were estimated from bulk host transcriptome data using the CIBERSORT X algorithm.<br><br>scRNAseq: Raw sequencing reads were aligned to GRCh38 using the STAR aligner. Cell barcodes were then determined based upon UMI count distribution. Read count matrices were generated through the 10X genomics cellranger pipeline v3.0. Data was processed and analyzed using Scanpy v1.6. Cells that had <200 genes and had greater than 30,000 counts were filtered. Mitochondrial genes were removed and multi-sample integration was performed using Harmony v0.1.4.<br><br>SARS-CoV2 viral load by mNGS: All samples were processed through a SARS-CoV-2 reference-based assembly pipeline that involved removing reads likely originating from the human genome or from other viral genomes annotated in RefSeq with Kraken2 v.2.0.8_beta, and then aligning the remaining reads to the SARS-CoV-2 reference genome MN908947.3 using minimap2 v.2.17. We calculated SARS-CoV-2 reads-per-million (rpM) by dividing the number of reads that aligned to the virus with mapq≥20 by the total number of reads in the sample (excluding reads mapping to ERCC RNA standards).<br><br>Figures were made using the pheatmap v1.0.12 and ggplot2 v3.3.3 packages for R and GraphPad Prism. Code for the differential expression and cell type proportions analysis is available at: https://github.com/AartikSarma/COVIDARDS.

For manuscripts utilizing custom algorithms or software that are central to the research but not yet described in published literature, software must be made available to editors and reviewers. We strongly encourage code deposition in a community repository (e.g. GitHub). See the Nature Research guidelines for submitting code & software for further information.

Data

Policy information about availability of data

All manuscripts must include a data availability statement. This statement should provide the following information, where applicable:

- Accession codes, unique identifiers, or web links for publicly available datasets
- A list of figures that have associated raw data
- A description of any restrictions on data availability

The raw sequencing data are protected and are not available due to data privacy restrictions from the IRB protocols governing patient enrollment in this study. The processed genecount data are available from the National Center for Biotechnology Information Gene Expression Omnibus database under accession code GSE163426 [https://www.ncbi.nlm.nih.gov/geo/query/acc.cgi?acc=GSE163426]. The published human lung single-cell datasets53 used for cell type proportions analysis can be obtained through Synapse under accessions syn21560510 [https://www.synapse.org/#!Synapse:syn21560510] and syn21560511 [https://www.synapse.org/#!Synapse:syn21560511].

Field-specific reporting

Please select the one below that is the best fit for your research. If you are not sure, read the appropriate sections before making your selection.

☒ Life sciences ☐ Behavioural & social sciences ☐ Ecological, evolutionary & environmental sciences

For a reference copy of the document with all sections, see nature.com/documents/nr-reporting-summary-flat.pdf

Life sciences study design

All studies must disclose on these points even when the disclosure is negative.

Sample size	Samples were selected from an observational cohort. We used the RNASeqPower package for R to calculate the power of differential expression analysis, and determined that we had greater than 99% to detect a 2-fold change in expression at an FDR < 0.1 in our primary analysis.
Data exclusions	Inclusion criteria were: 1) admission to the intensive care unit for mechanical ventilation for ARDS or airway protection, 2) age ≥ 18 years, 3) availability of TA collected within five days of intubation yielding 106 protein-coding transcripts by RNA-seq.<br>Exclusion criteria were: 1) withdrawal of consent, 2) evidence of LRTI but no ARDS, 3) no TA specimen available within five days of intubation, 4) TA specimen yielding < 10^6 protein-coding transcripts by RNA-seq, 5) receipt of immunosuppressive medication or underlying immunocompromising condition prior to tracheal aspirate collection.<br>Of the 360 enrolled subjects, 86 had ARDS, clinical evidence of lower respiratory tract infection (LRTI), or no radiographic evidence of pulmonary disease, and also had TA samples available for RNA-seq. Subjects who withdrew consent (n=1), who had LRTI but no ARDS (n=-2), who did not have a TA sample collected within five days of intubation (n=7), or who had TA samples yielding < 1x106 protein-coding transcripts on RNA-seq (n=1), were excluded. Of the remaining 75 eligible subjects, those treated with immunosuppression or with underlying immunocompromising conditions were excluded, leaving 15 COVID-ARDS, 32 Other-ARDS and five No-ARDS subjects for final analysis.
Replication	All analyses were performed in a single cohort of patients. We have made a concerted attempt to clearly indicate the number of patients analyzed in each comparator group (COVID-ARDS, Other-ARDS, No-ARDS) in the manuscript and figure legends. This is the first publicly available sequencing dataset of tracheal aspirates from patients with ARDS, and there is therefore no dataset available for a replication analysis.

Randomization	This is an observational study comparing tracheal aspirate gene expression based on ARDS etiology (COVID vs. Other), and thus randomization into intervention groups is not applicable.
Blinding	Investigators were blinded to any information about gene expression or metagenomic sequencing prior to chart review for phenotype assignment. Samples from both studies were sequenced together, and the sequencing and alignment pipeline did not have any information about the subject diagnosis.

Reporting for specific materials, systems and methods

We require information from authors about some types of materials, experimental systems and methods used in many studies. Here, indicate whether each material, system or method listed is relevant to your study. If you are not sure if a list item applies to your research, read the appropriate section before selecting a response.

Materials & experimental systems

n/a	Involved in the study
☒	☐ Antibodies
☒	☐ Eukaryotic cell lines
☒	☐ Palaeontology and archaeology
☒	☐ Animals and other organisms
☐	☒ Human research participants
☒	☐ Clinical data
☒	☐ Dual use research of concern

Methods

n/a	Involved in the study
☒	☐ ChIP-seq
☒	☐ Flow cytometry
☒	☐ MRI-based neuroimaging

Human research participants

Policy information about studies involving human research participants

Population characteristics	We included 15 patients with COVID ARDS. The median age of these patients was 54.8, and they were more likely to be male (60%) and Hispanic (53.3%). We compared these patients to 32 patients with ARDS. The median age of these patients was not significantly different (61.4, p = 0.205), and a similar proportion was male (62.5%, p = 1.000). A smaller proportion was Hispanic (9.4%, p = 0.003), reflecting the disproportionate impact of the COVID-19 pandemic on minority communities in the United States. We also included 5 mechanically ventilated controls. Again, there was no difference in age (66.2, p = 0.190) or sex (40% male) compared to the COVID ARDS patients. No control patient identified as Hispanic, but this difference did not meet statistical significance (p = 0.114). For complete demographic details, please see Table 1.
Recruitment	For both the COVID-19 and control cohorts, if a patient met inclusion criteria, then a study coordinator or physician obtained written informed consent for enrollment from the patient or their surrogate. Patients or their surrogates were provided with detailed written and verbal information about the goals of the study, the data and specimens that would be collected, and the potential risks to the subject. Patients and their surrogates were also informed that there would be no benefit to them from being enrolled in the study and that they may withdraw informed consent at any time during the course of the study. All questions were answered, and informed consent documented by obtaining the signature of the patient or their surrogate on the consent document (or during the COVID-19 pandemic, the IRB-approved electronic equivalent, to enable touchless consent).<br>Many critically ill patients are unconscious at the time of intensive care unit (ICU) admission due to their underlying illness and/or are endotracheally intubated for airway management or acute respiratory failure. The patients who are not unconscious are often in pain and may have acute delirium due to critical illness and/or medications. For these reasons, many subjects are unable to provide informed consent at the time of enrollment. Because this study could not practically be done otherwise and was deemed to be minimal risk by the UCSF IRB, if a patient was unable and a surrogate was not available to provide consent, patients were enrolled with waiver of initial consent, including the collection of biological samples.<br>Specifically, for subjects who were unable to provide informed consent at the time of enrollment, our study team was permitted to collect biological samples as well as clinical data from the medical record obtained prior to consent. Surrogate consent was vigorously pursued for all patients; moreover, each patient was regularly examined to determine if and when s/he was able to consent for him/herself, and the nursing and ICU staff were contacted daily for information about surrogates' availability. For patients whose surrogates provided informed consent, follow-up consent was subsequently obtained from the patient if they survived their acute illness and regained the ability to consent. For subjects who died prior to the consent being obtained, a full waiver of consent was approved by the UCSF IRB for both cohort studies. Lack of a surrogate to provide consent is common in critically ill patients. To address this, the UCSF IRB also approved a full waiver of consent for subjects in the COVID-19 cohort who remained unable to provide informed consent and had no contactable surrogate identified within 28 days. Before utilizing this waiver, we made and documented at least three separate attempts to identify and contact the patient or surrogate over a month-long period. While most patients enrolled were consented by typical processes, nine died prior to consent being obtained, and three were included with a full waiver of consent due to lack of ability to consent and lack of contactable surrogate. No personally identifiable information has been included as part of this manuscript for any enrolled patients.
Ethics oversight	We studied patients who were enrolled in either of two prospective cohort studies of critically ill patients at the University of California, San Francisco (UCSF) and Zuckerberg San Francisco General Hospital. Both studies were approved by the UCSF Institutional Review Board under protocols 17-24056 and 20-30497, respectively, which granted a waiver of initial consent for tracheal aspirate and blood sampling as described above.

Note that full information on the approval of the study protocol must also be provided in the manuscript.
